# How Is Existential Threat Related to Intergroup Conflict? Introducing the Multidimensional Existential Threat (MET) Model

**DOI:** 10.3389/fpsyg.2016.01877

**Published:** 2016-12-05

**Authors:** Gilad Hirschberger, Tsachi Ein-Dor, Bernhard Leidner, Tamar Saguy

**Affiliations:** ^1^School of Psychology, Interdisciplinary Center HerzliyaHerzliya, Israel; ^2^Department of Psychology, University of Massachusetts Amherst, AmherstMA, USA

**Keywords:** existential threat, intergroup conflict, political attitudes

## Abstract

Existential threat lies at the heart of intergroup conflict, but the literature on existential concerns lacks clear conceptualization and integration. To address this problem, we offer a new conceptualization and measurement of existential threat. We establish the reliability and validity of our measure, and to illustrate its utility, we examine whether different existential threats underlie the association between political ideology and support for specific political policies. Study 1 (*N =* 798) established the construct validity of the scale, and revealed four distinct existential threats: personal death (PD), physical collective annihilation (PA), symbolic collective annihilation (SA), and past victimization (PV). Study 2 (*N* = 424) confirmed the 4-factor structure, and the convergent and discriminant validity of the scale. Study 3 (*N* = 170) revealed that the association between a hawkish political ideology and support for hardline policies was mediated by PV, whereas the association between a dovish political ideology and conciliatory policies was mediated by concerns over collective symbolic annihilation. Study 4 (*N* = 503) conceptually replicated the pattern of findings found in Study 3, and showed that at times of conflict, PA concerns also mediate the relationship between hawkish ideologies and support for hardline policies. In both Studies 3 and 4, when controlling for other threats, PD did not play a significant role. These results underscore the need to consider the multidimensional nature of existential threat, especially in the context of political conflict.

## Introduction

In the context of intergroup conflicts, the motivations for both violence and for reconciliation are driven to a large extent by existential concerns (Pyszczynski et al., 2003; Bar-Tal, 2007; Hirschberger and Pyszczynski, 2010). Whereas much work has considered such threats to be rooted in personal fear of death (e.g., Pyszczynski et al., 2003), others have conceptualized existential threats as representing a collective-level concern for the in-group’s future existence (e.g., Wohl et al., 2010). Although useful, each of these frameworks relies on a relatively narrow conceptualization of existential threats, focusing solely on individual-level processes, or solely on collective processes. These conceptualizations further overlook additional dimensions pertaining to the nature of the threat (being realistic or symbolic; Stephan and Stephan, 2000), and to its temporal orientation (stemming from the past vs. forward-looking). Although there are several noteworthy attempts in the literature to consider more than one, singular existential threat (e.g., the meaning maintenance model; Heine et al., 2006), which enables a more nuanced understanding of the role of threat in political conflict (Rovenpor et al., 2016), to date, a theoretically driven integration of these different dimensions of existential threat is missing. The goal of the present research, is to first and foremost develop a novel, multidimensional conceptualization of existential threat and determine whether different existential threats are related to different political outcomes in a theoretically meaningful way. To do so, we first established the psychometric properties (reliability, validity) of our model and then demonstrated the viability of this new multidimensional construct in answering longstanding questions on the relationship between ideology and policy support. We contend that a more nuanced understanding of existential threat would promote a more complex understanding of the relationships between threat, ideology, and political attitudes than currently exists in the literature.

### Existential Threat

Although the term *existential threat* is used frequently in the psychological literature, it is an elusive term that can mean different things, and there is no one agreed upon definition of what constitutes an existential threat. An existential threat, put simply, is a threat to survival (May et al., 1958), but this narrow definition does not suffice because the survival of a person or a group is not only defined by their physical existence, but also by their ability to maintain their identity – their sense of self. Thus, a person with advanced dementia exists according to a narrow definition of existence, but is no longer the person he or she used to be if we include identity as an integral aspect of being. Similarly, a group may experience existential threat if it is concerned that another group might annihilate it, and also when its culture, symbols and beliefs are threatened to the point that the group might transform and change into another unrecognizable entity. Importantly, existential threat is a *perceptual phenomenon*, meaning that a veritable threat to existence does not have to be present for someone to experience a sense of existential threat. Often, there is a dialectical relationship between the perception of threat and the actual existence of a threat, but this gap between perception and reality leaves much room for individual variation – different people in the same situation may perceive the threat in a different manner. In the current paper we argue that these individual differences in the perception of threat are an important yet unexplored phenomenon. We demonstrate that a multidimensional model of existential threats is reliable and valid, and illustrate the utility of this model in understanding intergroup conflict. Specifically, we show that understanding existential threat as a multidimensional phenomenon may shed light on the psychological processes underlying adherence to specific political ideologies.

### Current Models of Existential Threat: Terror Management

The study of existential political cognitions has been primarily conducted along three distinct paths: Terror management theory (TMT: Greenberg et al., 1997), collective future-oriented existential concerns (e.g., Wohl et al., 2010), and collective victimization, an existential concern that is primarily past-oriented (e.g., Noor et al., 2012; Vollhardt, 2012, 2015; Schori-Eyal et al., 2014). TMT (Greenberg et al., 1997) is arguably the most theoretically elaborate attempt to date to understand how existential concerns shape social cognitions and behaviors. The theory posits that the juxtaposition of an instinctive desire for continued life with the uniquely human awareness of the inevitability of death creates a potential for paralyzing terror that is managed by adherence to a cultural worldview. From the perspective of TMT, because consensual validation of worldviews and self-conceptions are needed for effective protection against anxiety, the mere existence of those with different worldviews is threatening. Terror management studies support these postulations and show that priming thoughts of personal death (MS: mortality salience) increase the motivation to invest in one’s worldview, as well as to avoid, derogate, punish, and even aggress against worldview-threatening others (e.g., Greenberg et al., 1990; McGregor et al., 1998; Hirschberger and Ein-Dor, 2006; Pyszczynski et al., 2006).

Research conducted over the past decade has documented the impact of death concerns on political attitudes, and on the preference for violent solutions to conflict, and has shown that MS leads conservative Americans to support military operations in fighting terrorism (Pyszczynski et al., 2006), right-wing Israelis to support violent resistance against policies that threaten their worldview (Hirschberger and Ein-Dor, 2006), Israelis to support a pre-emptive nuclear attack on Iran (Hirschberger et al., 2009), Israeli Jews, Arabs, and South Koreans to resort to a retributive justice-related mindset rather than to a rational utility mindset when considering intergroup conflict (Hirschberger et al., 2015, 2016b), Americans to display implicit negativity toward Arabs and immigrants (Motyl et al., 2011), and Iranians to increase their support for suicide bombing to fight the US and its allies (Pyszczynski et al., 2006). Meta analyses have validated the effects of mortality salience and established its uniqueness compared to alternatives such as uncertainty and meaning (Burke et al., 2010).

Despite this solid body of evidence, one limitation associated with terror management research is its almost exclusive focus on existential fear at the individual-level. Indeed, the theory does not clearly distinguish between individual-level and collective-level death anxiety, sometimes treating the two as interchangeable. For example, some terror management studies have replaced the typical MS prime with reminders of collective-level events such as “9/11” (Landau et al., 2004) or the Palestinian Nakba (i.e., catastrophe in Arabic, referring to the 1948 war; Hirschberger et al., 2016b). Further, TMT refers to symbolic collective identity as the antidote to death anxiety because the collective self is likely to survive the death of the physical self (Solomon et al., 2015). Although terror management research has shown that threatening these symbolic collective structures elicits elevated death-related cognitions (Schimel et al., 2007), there is no attempt, as of yet, to distinguish between threats to the individual self and threats to the collective self. We contend that despite the expected similarities in responses to individual-level and collective-level threats, there may also be important differences between these threats, and the complex dynamics between them, that are overlooked in terror management research. Currently, no theoretical model can account for such possibilities.

### Collective Existential Threat

With respect to threats at the collective level, research on collective angst (Wohl et al., 2010) addresses some of the limitations of TMT. Collective angst contributes to the understanding of existential threat and political conflict by conceptualizing threat at the group level and examining how angst serves as a mediator between threats to the group and various intra- and intergroup outcomes. The collective angst literature, however, does not clearly distinguish between symbolic and concrete concerns, and is also inconsistent in the definition and measurement of collective angst, with different items measuring this construct in different publications. In most studies it seems to reflect a symbolic concern over loss of collective identity (e.g., “I think the future of the American way of life is under threat from abroad,” Wohl and Branscombe, 2009; “I feel secure about the future of Kansas University,” Wohl et al., 2010), whereas in other studies (e.g., Halperin et al., 2013), collective angst seems to reflect a combination of realistic (“I feel confident that Israel will survive”) and symbolic (“I feel that Israel will always thrive”) concerns. The current research proposes a more nuanced conceptualization of collective existential threats that examines the differences between concrete and symbolic threats.

Such distinction between concrete (i.e., realistic) and symbolic threats has been previously proposed by Stephan and Stephan (2000) in their integrated threat theory, which presents a solid basis upon which to build a multidimensional conceptualization of existential threats in the context of intergroup relations. The theory contends that a threat is present anytime “one group’s actions, beliefs, or characteristics challenge the goal attainment or well-being of another group” (Riek et al., 2006, p. 363). It distinguishes between two types of threat: realistic threat – when an adversarial group presents a risk to the safety, economy, politics, health or well-being of the ingroup – and symbolic threats that primarily involve “perceived group differences in morals, values, standards, beliefs, and attitudes” (Oskamp, 2000, p. 42). Over the years, the theory was further elaborated to include the distinction between threats to the group as a whole, and personal threats to individual members (Stephan and Renfro, 2002). Despite its multidimensional perspective, integrated threat theory does not distinguish between relatively benign threats (such as common political and economic threats), and threats to the very existence of the group.

### Past and Future Threats

Another element that seems to be lacking in current conceptualizations of threat has to do with the temporal nature of the threat. A growing number of studies have pointed to threats stemming from a sense of collective victimization (e.g., Vollhardt, 2012). Collective victimization is a complex term that has been defined as the experience of undeserved, immoral and intentional harm inflicted on the group by another group (Bar-Tal et al., 2009). This perception of harm may have implications on several levels of analysis: The macro level (society); the meso level (group and intergroup); and the micro-level – the individual (Vollhardt, 2012). Further, collective victimization may refer to historical victimization that leaves a traumatic memory, and may also refer to victimization in a contemporary conflict that is ongoing and does not necessarily stem from a history of victimization (Schori-Eyal et al., 2014). In the current research we limit our discussion of collective victimization to the collective implications of historical victimization. Historical victimization is considered a fundamental level of collective victimhood (Schori-Eyal et al., 2014) in contexts in which there is a history of collective trauma. Historical victimization is relevant to understanding intergroup conflict because often the pains and woes of past events are conflated with contemporary conflict (Lifton, 2005); instill a sense of siege among victims (Bar-Tal and Antebi, 1992; Hirschberger et al., in press); and foster a belief in perpetual ingroup victimization (Schori et al., 2009), which may ironically increase as time from the historical trauma passes (Klar et al., 2013).

Research demonstrates that priming a group’s past victimization (PV) elicits in-group protective behaviors (Wohl and Branscombe, 2008), greater adherence with the group’s ideology and narrative (Canetti et al., in press), and more belligerence toward out-groups (Hirschberger et al., in press). This effect of victimization on conflict is not uniform, however, with exclusive victim beliefs usually associated with negative attitudes toward out-groups, and inclusive beliefs with more forgiveness and reconciliation motivations (Vollhardt, 2013, 2015; Vollhardt et al., 2016; but see Cohrs et al., 2015 for a more nuanced understanding of inclusive beliefs). The research on PV and collective angst begs the question: are these two constructs redundant? In other words, are all collective existential threats one and the same, or is the temporal orientation of the collective threat significant? Some research seems to suggest that collective threats elicit similar reactions, whether they are emanating from collective memory or from anticipation of the future (Wohl and Branscombe, 2008). In the current research we examine the possibility that there may be important differences between future- and past- oriented collective concerns. Although both of these threats ultimately influence perceptions of the future, when the lens through which one looks is shaped by a memory of collective trauma it is more likely that this perception will be contaminated with a sense of perpetual victimhood (Klar et al., 2013; Canetti et al., in press). Conversely, an onward-looking perception of threats that is not burdened with collective memory may be more attuned to reality, and resistant to the biasing effects of narrative.

### The Multidimensional Existential Threat (MET) Model

Taken together, the extant literature on existential concerns calls for a more nuanced conceptualization of threats, considering their temporal nature, their key target (self vs. group), and also their realistic versus symbolic nature. On this basis, we propose the MET model that conceptualizes existential threats as ranging along three axes: individual-collective, physical-symbolic, and future-oriented past-oriented. The current research takes the different existential threats delineated in the literature and examines whether there is support for a multidimensional model of threats over a unitary conceptualization of threat (i.e., all threats are one and the same). After we establish the construct validity of the MET model, we examine the convergent and discriminant validity of existential threats by examining their associations with other related psychological constructs. Finally, to demonstrate the necessity of a multidimensional model of threats in the intergroup literature, we examine whether different existential threats can help understand the relationship between political ideology and specific policy preferences.

It is important to note that in this research we restrict our investigation to those threats that have been identified in the literature on existential threat and political conflict: TMT; collective existential threat; and past victimization. Because the literature on collective existential threats confounds physical and symbolic threats, in the current research we attempt to tease those apart, enabling us to test four different existential threats that emanate from the extant literature. Clearly, once existential threats are understood as a multidimensional construct it will be possible to expand the current model and extend the investigation of threats in other directions. It is also necessary to stress that this model is currently applied to a Jewish-Israeli context wherein physical and symbolic collective threats are perceived as central to the conflict, and where the long-term victimization and statelessness of the Jewish people has a long-term impact that reverberates in the present (e.g., Klar et al., 2013; Canetti et al., in press).

On this basis, we extract four distinct existential threats that are relevant to intergroup relations: (a) An individual future-oriented physical threat – personal death (PD: e.g., “I am troubled by thoughts of my own death”); (b) A collective future-oriented physical threat – physical collective annihilation (PA: e.g., “The existence of my people is in jeopardy”); (c) a collective future-oriented symbolic threat – symbolic collective annihilation (SA: e.g., “Eventually, my people will assimilate into other cultures”); and a collective past-oriented threat – past victimization (PV: e.g., “I tend to reflect on the difficult history of my people”).

Personal death is an individual-level physical threat that is future oriented and constitutes the knowledge that death is inevitable, unpredictable and irreversible. TMT is the main theoretical perspective that focuses on this type of existential threat and predicts that as there is no solution to physical death, people will resort to symbolic defenses such as worldview defense (e.g., Greenberg et al., 1997). Collective existential threats have not been well-defined thus far, with collective angst amorphously situated between physical and symbolic threats. In the current research we set out to clearly distinguish between these two types of collective existential threat. Collective physical annihilation constitutes a threat to the physical existence of the group – for example, the Israeli fear of Iran’s nuclear program (Ben Meir and Shaked, 2007). Unlike the physical destruction of the individual, however, the physical destruction of the group is not inevitable, and can be prevented. For this reason, we expect collective existential threats to exert significantly different effects than personal death, and to promote a hypervigilant cognitive system that is ready to prevent threats, or confront them, rather than deny them.

Symbolic collective annihilation constitutes the fear that the group will lose its identity in the future (i.e., optimal distinctiveness; Brewer, 2003) and will transform into an entity different than what it is today – for example the fear among some Israelis that Israel will be replaced by a bi-national state and will, thus, lose its Jewish identity, or the fear among some Europeans that concerns over terrorism may lead to a restriction of civil liberties in Europe. Symbolic and physical threats sometimes go hand in hand because when the group is physically destroyed the culture will be destroyed as well, but, these threats may also reflect conflicting concerns. For example, Israel’s continued occupation of the West Bank, with its large Palestinian population, poses a threat to Israel’s identity as a Jewish state and a democracy. Ceding control over these territories, however, while alleviating this symbolic concern may simultaneously elevate individual-level concerns for personal safety. A multidimensional model of existential threats enables us to highlight these differences, and provides a lens through which we may better understand the existential dilemma between seemingly incongruent threats.

A sense of existential threat may stem not only from a forward-oriented perspective looking into the future and identifying possible threats. It may, to a large extent, be motivated by the group’s history such that groups that were victims of collective trauma may show amplified vigilance and caution when assessing contemporary challenges and threats. This component of existential threat, which is a sub-category of collective victimization (e.g., Noor et al., 2012; Vollhardt, 2012), accounts for how representations of history impact contemporary political cognitions (Imhoff et al., 2016). It reflects the centrality and chronic salience of victimhood, but does not account for other aspects of victimhood such as the severity of in-group victimization (Vollhardt, 2012), the framing of victimhood beliefs (Vollhardt, 2015), or competitive victimhood (Noor et al., 2012).

Past traumas are stored in collective memory as a mindset – a prism through which the group views the world and analyzes new information (Bar-Tal et al., 2009). Under this mindset, group members are prone to view themselves as victims in new situations that are perceived as threatening. Research indicates that thinking about past collective traumas reduces feelings of collective guilt toward the in-group’s actions (Wohl and Branscombe, 2008), and reduces the acknowledgment of violence committed by the group (Čehajić and Brown, 2010; Hirschberger et al., 2016a). In the context of the Israeli–Palestinian conflict, Halperin and Bar-Tal (2011) found that beliefs about collective victimhood reduce support for compromises. These patterns of responses suggest that higher perceptions of past victimization will be associated with more belligerent reactions toward current adversarial out-groups.

Thus, it seems that personal death and past victimization deal with inevitable outcomes – those that have occurred in the past or that will occur with certainty in the future. As such, they are hypothesized to be associated with negative and hostile reactions to adversary groups. The reactions that stem from these two types of threats, however, are not necessarily the same. Personal death is associated with intergroup aggression as a means of affirming one’s symbolic worldview (e.g., Pyszczynski et al., 2006; Hirschberger et al., 2009), whereas past victimization is associated with aggression under the dictum of *never again*. This historical mindset, however, could be framed in inclusive, not exclusive terms (Vollhardt, 2013, 2015; Cohrs et al., 2015), and then reflecting on the group’s past may be associated with less violent inclinations.

In contrast to threats of personal death and past victimization, collective physical threats and collective symbolic threats address existential concerns that are possible, but not certain (the role of uncertainty in the effects of existential threat has been a source of controversy; Burke et al., 2010; Hogg, 2014). As such, they induce a preventative motivation unique to each perceived threat – at times the two will act in unison, such as when both existence and identity are in jeopardy, and at other times they may reflect antagonistic motivations – one relating to belligerent and the other to peaceful motivations. This preventative motivation depends to a large degree on the context and primarily on the perceived ability to avoid the threat, or remove the threat by force. When the threat can be avoided or reduced in a non-violent manner, these means will be preferred. When violence seems inevitable, however, it will be condoned as long as it serves the goal of group preservation (e.g., Hirschberger et al., 2009).

## The Present Research

The goals of the present research were threefold: First, in Study 1 to establish the construct validity of our four-factor conceptualization of existential threats. Then, in Study 2, to confirm the structure of the scale and compare the four-factor model with other possible theoretically relevant solutions. In this study we also set out to examine the convergent and discriminant validity of the scale. The third and final goal was to examine whether existential threats mediate the relationship between political orientation, in-group identity and specific policy attitudes on two samples of Israeli Jews – one collected at a time of relative calm in Israel (Study 3), and the other immediately following the 2014 Israel–Gaza War (Study 4). Thus, Study 3 measured general attitudes toward Palestinians and support for peace, and Study 4 focused specifically on attitudes pertaining to the aftermath of the 2014 Israel–Gaza War. All of the research presented here was approved by the IRB of the Interdisciplinary Center.

## Study 1

The purpose of Study 1 was to construct a measure of existential threats based on our theoretical postulations, and examine whether this measure produces the four distinct hypothesized factors: personal death, collective physical annihilation, collective symbolic annihilation, and past victimization.

### Method

#### Participants

A community convenience sample (*N* = 798), 438 men and 360 women ranging in age from 18 to 72 (*M* = 34.00, *SD* = 16.27) was recruited on commuter trains running between Tel-Aviv and Haifa (center and north of Israel, respectively). Research assistants approached commuters and asked them whether they would be willing to answer a short survey. Those who agreed (64% response rate), were given a questionnaire packet that was collected 20–25 min later.

#### Materials

The questionnaire included items assessing the MET scale. We drafted items for the four dimensions that are relevant to intergroup conflict: personal death, collective physical annihilation, collective symbolic annihilation, and past victimization. Prior to collecting data, the initial scale included 50 items that were drafted by the authors and two of their graduate students. The instructions for drafting the items was to come up with simple statements that convey the idea of each theoretical facet of the MET while clearly distinguishing it from the other facets. Thus, personal death (PD) should include items that discuss an individual, not collective threat; collective physical annihilation (PA) and symbolic annihilation (SA) should include items that reflect collective threat that is future oriented with a clear distinction between threats to the physical existence of the group and threats to its character and identity. Past victimization (PV) was to include items reflecting the memory of collective trauma. Then, two independent judges were given a brief explanation on the four MET factors, and were asked to sort the 50 items into the four categories and to note whether some items could be included in more than one category, and whether items were redundant with others. Disagreements between judges were resolved through consensus. This process resulted in the removal of 19 items (e.g., items such as “I am worried about the future of Israel” – a collective angst item, were removed because they were not specific enough to distinguish between symbolic and physical concerns). The remaining scale comprised 31 items. Six of the items tapped the PD dimension; Seven items tapped the PA dimension; Ten items tapped the SA dimension; and eight items tapped the PV dimension. Items were answered on seven-point scales ranging from “1” strongly disagree to “7” strongly agree. After completing the scale, participants answered a brief demographic sheet and were debriefed by the research assistant collecting the questionnaire.

### Results and Discussion

To determine the ideal number of factors for the MET scale, we employed a parallel analysis – a precise method for determining the number of factors to retain in factor analysis (Ledesma and Valero-Mora, 2007). The analysis indicated that in keeping with our hypothesis, the most efficient number of factors for the MET scale is four (the scree plot concurs with this conclusion). Next, we used Winsteps software^1^, a program designed to help develop scales by providing in depth examination of questionnaire items such as the distinctiveness of items, and the variance of each item to create optimal spread of responses across the scale. In the current study, we used this software to estimate the effectiveness of each item in distinguishing between participants, as well as to locate items with multiple loadings. During this process, we dropped 11 items from the MET scales – 4 because of multiple loadings, and 7 because of low discriminant power – such that in the final 20-item scale six items assessed SA, seven items assessed PA, four items assessed PV, and three items assessed PD. **Table 1** presents a full list of items, item-loadings based on Exploratory Factor Analysis (EFA), and reliabilities (one should note that because many of the SA items are presented in reversed form, e.g., “Jewish culture is eternal” it less intuitive to think of this factor as a measurement of threat). To verify the results of the EFA and to examine the convergent and discriminant validity of the MET, we conducted Study 2.

**Table 1:** Item loading of 4-factor model factors (EFA; Study 1).

Item	SA	PA	PV	PD
The Jewish people are an eternal people	-0.90			
I believe that Jewish identity will always exist	-0.81			
Jewish culture is eternal	-0.79			
Jewish culture will always maintain its uniqueness	-0.84			
Jewish culture will always be distinct from other cultures	-0.58			
Eventually, Jews will assimilate into other cultures	0.61			
The physical existence of the Jewish people is in danger		0.64		
The State of Israel is on the verge of annihilation		0.65		
The existence of the Jewish people is in jeopardy		0.78		
The future for the Jews looks black		0.74		
The State of Israel faces a threat to its existence		0.59		
The State of Israel will not survive for very long		0.49		
There is no real threat to the existence of the Jewish people		-0.56		
I often think of the grievances caused to my people in the past			0.87	
The negative treatment of my people in the past bothers me			0.78	
I tend to reflect on the difficult history of my people			0.78	
I usually do not think about my people’s negative history			-0.50	
I often think that my life is in danger				0.74
I am troubled by thoughts of my own death				0.50
My existence is in jeopardy				0.78
*Reliability*	0.83	0.80	0.82	0.71

## Study 2

The purpose of Study 2 was first, to verify Study 1’s EFA by means of a Confirmatory Factor Analysis (CFA). Second, to compare the four-factor solution to other possible solutions that make theoretical sense: a one-factor solution combining all existential threats into a single factor, and a three-factor solution in which collective and individual physical threats are collapsed into one factor. The one factor solution accounts for the possibility that our theoretical postulations are incorrect and that all existential threats are one and the same. In this case, there would be no need for a multidimensional scale. The three-factor solution addresses the possibility that there is no differentiation between physical existential threats, and that individual-level threat and collective-level threats are not distinct. This possibility stems from research showing that primes of personal death and collective threats elicit similar effects [i.e., the similar effects of MS (e.g., Pyszczynski et al., 2006) and collective angst (e.g., Wohl et al., 2010) on political attitudes]. If the four-factor solution will have better fit than these theoretically reasonable alternatives, we can proceed to study existential threats as a multidimensional construct with greater confidence.

Third, we designed Study 2 to examine the convergent and discriminant validity of the MET. To do so, we examined associations between MET factors and other theoretically relevant constructs. We measured collective angst (Wohl et al., 2010) to determine whether angst primarily reflects physical or symbolic collective concerns; moral justification (Bandura et al., 1996) to determine whether individual or collective existential concerns are related to relaxed moral standards; fear of personal death (Lester and Abdel-Khalek, 2003), neuroticism (John et al., 1991), and positive and negative affect (Watson et al., 1988) to determine that MET factors are not merely expressions of neuroticism, affect, or death anxiety; in-group identification (with attachment and glorification subscales; Roccas et al., 2006) and religiosity to examine whether ideologies and worldviews distinguish between MET factors; and essentialism (Bastian and Haslam, 2006) to rule out the possibility that differences between MET factors can be explained by essentialism. Because MET items reflect general concerns about physical and symbolic threats (i.e., the future of the people or the culture) and not the specific content of current threats, we constructed a scale that taps specific physical (e.g., the threat of war and terrorism) and specific symbolic (e.g., a deterioration in the level of freedom in society) threats. Further, the MET asks about the level of perceived threat and not the estimated likelihood that the threat will manifest itself. We, therefore, asked participants to estimate the risk of physical and symbolic existential threats in the near future. Together, these scales constitute a wide spectrum of emotions, worldviews, perceptions and cognitions that allow a careful examination of the unique attributes of each MET factor.

### Method

#### Participants

A near-representative sample of Israeli Jews matched for age and gender (*N* = 424), 205 men and 219 women ranging in age from 18 to 70 (*M* = 40.11, *SD* = 14.79) was recruited through iPanel^2^, an online panel consisting of over 100,000 participants representing every geographic and demographic sector of Israel within the 1967 borders. 58.8% of participants completed the questionnaire in full.

#### Materials and Procedure

Participants completed the following scales in randomized order: Fear of personal death was measured with the seven items from the Collett-Lester fear of death scale (Lester and Abdel-Khalek, 2003; e.g., “the shortness of life” α = 0.91) that tap fear of personal death (the other factors tap fear of dying and fear of others’ death). Items were answered on a 7-point scale ranging from (1) “not at all concerned” to (7) “very concerned”; The 20-item MET as in Study 1 [MET SA (α = 0.88), MET PA (α = 0.85), MET PV (α = 0.82), MET PD (α = 0.77)]; A shortened eight-item in-group identification scale (Roccas et al., 2006), with four attachment (e.g., “Being an Israeli is an important part of my identity”; α = 0.88) and four glorification items (e.g., “Relative to other nations, we are a very moral nation”; α = 0.77), answered on a seven-point scale ranging from (1) “strongly disagree” to (7) “strongly agree”; A five-item collective angst scale (Wohl et al., 2010; e.g., “I feel that Israel will always thrive” α = 0.87) answered on a seven-point scale ranging from (1) “strongly disagree” to (7) “strongly agree”; The 20-item positive and negative affect schedule (PANAS), with 10 positive (e.g., “excited”; α = 0.78), and 10 negative (e.g., “angry”; α = 0.83) items answered on a 7-point scale ranging from (1) “not at all” to (7) “very much.” An eight-item neuroticism scale (e.g., “I worry a lot” α = 0.77) answered on a 5-point scale ranging from (1) “strongly disagree” to (5) “strongly agree”; A 10-item moral justification scale based on Bandura et al. (1996) and adopted to the current culture and context (e.g., “when we need to defend ourselves, moral considerations are irrelevant”; α = 0.92) answered on a seven-point scale ranging from (1) “strongly disagree” to (7) “strongly agree”; An essentialism scale (Bastian and Haslam, 2006) comprising eight items that tap biological essentialism (e.g., “a person’s genetics explains to a large degree what kind of person he or she is; α = 0.79), and eight items that tap discreteness or trait essentialism (e.g., “either a person has certain traits or he or she doesn’t”; α = 0.62) answered on a 7-point scale ranging from (1) “strongly disagree” to (7) “strongly agree”; An eight-item scale constructed for the current research tapping concrete threats (e.g., “there is a serious threat of terrorism against our country”; α = 0.71), and symbolic threats (e.g., “our identity as a liberal democracy is in danger”; α = 0.81) answered on a 7-point scale ranging from (1) “strongly disagree” to (7) “strongly agree.” In addition, we asked participants to estimate the risk of an existential physical threat (“the very existence of the State of Israel is in danger in the near future”) and an existential symbolic threat (“the identity of Israel as a Jewish and democratic state is in danger in the near future”) on a scale ranging from (1) “very low risk” to (7) “very high risk.” Then, participants completed demographic information such as: age, gender, religiosity, and political orientation.

### Results

To verify Study 1’s EFA, we conducted a CFA in which we examined the fit of the four-factor solution model to the 20-item MET scale, and compared it with two alternative models: (1) a 3-factor model in which the items of PD and PA are loaded on one latent factor; and (2) a 1-factor model in which all of the MET items are loaded on a single latent factor. The effectiveness of each model was compared by the Sample-Size Adjusted Bayesian Information Criterion (Adjusted BIC). The model with the lowest adjusted BIC score is deemed as the most effective. The CFAs were estimated using MPlus 6.1 (Muthén and Muthén, 1998-2010) Structural Equation Modeling (SEM) software. Goodness of fit was examined by the Comparative Fit Index (CFI), Tucker Lewis Index (TLI), and Root Mean Square Error of Approximation (RMSEA) scores. The analyses indicated that in keeping with our hypothesis, the 4-factor model had the lowest adjusted BIC score (28885.16; as compared with the 3-factor model, 29335.30, and the 1-factor model, 31584.10), and reached an acceptable fit to the observed data, χ^2^_(139)_ = 465.12, *p* < 0.001, CFI = 0.93, *TLI* = 0.90, *RMSEA* = 0.07. Results of the 4-factor model are presented in **Table 2**.

**Table 2:** Item loadings of 4-factor model factors (CFA; Study 2).

Item	SA	PA	PV	PD
The Jewish people are an eternal people	0.86			
I believe that Jewish identity will always exist	0.65			
Jewish culture is eternal	0.88			
Jewish culture will always maintain its uniqueness	0.47			
Jewish culture will always be distinct from other cultures	0.79			
Eventually, Jews will assimilate into other cultures	0.85			
The physical existence of the Jewish people is in danger		0.76		
The State of Israel is on the verge of annihilation		0.77		
The existence of the Jewish people is in jeopardy		0.59		
The future for the Jews looks black		0.71		
The State of Israel faces a threat to its existence		0.82		
The State of Israel will not survive for very long		0.85		
There is no real threat to the existence of the Jewish people		0.33		
I often think of the grievances caused to my people in the past			0.87	
The negative treatment of my people in the past bothers me			0.88	
I tend to reflect on the difficult history of my people			0.79	
I usually do not think about my people’s negative history			0.43	
I often think that my life is in danger				0.55
I am troubled by thoughts of my own death				0.74
My existence is in jeopardy				0.83

To examine the convergent and discriminant validity of the MET, we conducted a series of regression analyses. In these analyses, MET factors served as the outcome measures (when predicting a specific factor, the other three factors were partialed out to obtain the unique predictors of each factor). Predictors were collective angst, positive and negative affect, neuroticism, moral justification, fear of death, biological essentialism, trait-like essentialism, in-group identification (with attachment and glorification subscales), perceived physical threat, perceived symbolic threat, risk assessment of a physical and symbolic threat, political orientation, and religiosity. Standardized coefficients and the percentage of explained variance are presented in **Table 3**. Tolerance scores revealed no indication for multi-co-linearity.

**Table 3:** Standardized coefficients for predicting MET factors.

	Outcomes			
Predictors	SA	PA	PV	PD
Collective angst	0.19ˆ***	-0.09	-0.02	0.03
Positive affect	-0.06	-0.04	0.04	-0.01
Negative affect	0.06	0.04	0.05	0.14ˆ*
Neuroticism	0.02	-0.05	0.03	0.12ˆ*
Moral justification	-0.08	-0.04	0.08	0.07
Fear of death	0.06	-0.12ˆ**	0.09	0.37ˆ***
Biological essentialism	0.06	0.02	0.05	-0.02
Trait-like essentialism	-0.06	0.05	-0.07	0.01
In-group attachment	-0.16ˆ**	-0.03	0.19ˆ**	-0.16ˆ**
In-group glorification	-0.08	0.00	-0.03	0.14ˆ**
Physical threat	-0.07	0.20ˆ***	0.01	-0.08
Symbolic threat	0.04	-0.05	-0.05	0.00
Estimate of physical threat	-0.03	0.36ˆ***	-0.01	-0.03
Estimate of symbolic threat	0.10ˆ*	0.11ˆ*	-0.06	0.06
Political orientation (higher is more right-wing)	-0.14ˆ**	0.08	0.02	-0.02
Religiosity (higher is more religious)	-0.11ˆ**	-0.05	0.13ˆ**	0.03
*R*-square	52.0%	44.9%	22.2%	47.3%

*^∗^*p* < 0.05, ^∗∗^*p* < 0.01, ^∗∗∗^*p* < 0.001.*

The analyses indicated that higher SA was linked with higher collective angst, lower in-group attachment, greater concerns over symbolic threats, a left-wing political orientation, and lower levels of religiosity. These associations support our predictions that symbolic existential concerns are higher among left-wing non-religious participants who are low in attachment to the State (perhaps because of the policies of the current government), but feel greater trepidation over the future character of the State. These results also confirm our suspicion that collective angst is ultimately a measure of collective, symbolic threats thus removing some of the ambiguity surrounding this construct.

Higher PA was related to higher perceived physical threats, concerns over both physical and symbolic threats, and lower fear of death [the zero-order correlation between PA and fear of death was positive *r*(423) = 0.24, *p* < 0.001, including the other MET factors as covariates suppresses this effect and reverses the direction of the relationship]. Higher PV was associated with higher in-group attachment and higher levels of religiosity indicating, as expected, that a position of victimization is a component of religious nationalism. Higher PD was linked with higher negative affect, neuroticism, fear of death and in-group glorification. Interestingly, fear of death was related to lower levels of in-group attachment, suggesting that the two facets of national identification have a differential relationship with fear of death. Overall, these associations provide convergent and discriminant validity to the MET. None of the MET factors were significantly predicted by the two essentialism scales or by moral justification.

The results of Study 2 confirm the structure of the MET that was found in Study 1 and provide evidence that our conceptualization of existential threats is a stable construct that replicates from sample to sample. Further, Study 2 supports our contention that existential threats are a multidimensional construct wherein different threats have different implications and are associated with different constructs. Our analyses indicate that each MET factor is uniquely predicted by different threats, concerns, and worldviews, and the clear distinction between MET factors allows us to proceed in our analysis of existential threats and political cognitions. Results also indicate that the constructs used to examine convergent and discriminant validity of the MET explained a large percentage of the variance (see **Table 3**). This validates our choice of constructs and the relevance of the MET to the issues measured by the constructs. To better understand how the four-factor MET model is associated with intergroup relations, we examined, in the next steps of our research, whether this conceptualization and measurement of existential threats may predict political outcomes in theoretically meaningful ways.

## Study 3

Studies 1 and 2 constitute an important step in confirming the construct, convergent, and discriminant validity of the MET, and provide a method to examine whether different existential threats are associated with different political outcomes. In Study 3, we examined whether the MET model could explain different political attitudes among Israelis regarding the Israeli–Palestinian conflict. Because this is the first study to examine the associations between MET factors and attitudes toward an out-group, we planned a gradual assessment of participants’ willingness to engage with the other group starting from perceptions of the variability of the out-group (Brauer and Er-rafiy, 2011), which constitutes a removed observation of the other group, going to trust toward and feelings of communality with out-group members, as well as willingness to compromise with the out-group for peace, which represent a higher level of engagement and a willingness to take considerable risks for the possibility of living together in peace, and thus go beyond mere perceptions of the out-group (Nadler and Liviatan, 2006; Saguy and Halperin, 2014).

We postulated that existential threats underlie the relationship between in-group identification and political attitudes. On the basis of the results of Study 2, we contended that Israeli Jews face two main existential threats: A physical collective threat – the fear that the State of Israel will be physically destroyed, and that political compromises may bring about its demise; and a symbolic collective threat – fear that the continued occupation of the Palestinians will eventually lead Israel to lose its identity as a Jewish State, a democracy, or both. Study 2 indicated an association between lower in-group identification and the collective symbolic association factor of the MET. In the current study we, therefore, expected that the association between in-group identification and political attitudes would be primarily mediated by concerns over both physical and SA with each one of these MET factors showing a different effect: PA would be associated with high in group identification and belligerent attitudes, whereas SA would be associated with low identification and peaceful attitudes. We further postulated that these effects would occur independently after controlling for the other MET factors. Because previous research has indicated that both PD and PV are associated with greater hostility toward out-groups (Hirschberger et al., 2009; Halperin and Bar-Tal, 2011), we predicted that these two MET factors would also mediate the association between in-group identification and hawkish political attitudes. It should be noted that research often shows that existential threat is the driving force of nationalism and not the underlying mechanism explaining the link between nationalism and militancy (e.g., Castano, 2004; Canetti et al., in press). In the current research, however, we suggest that because in-group identification is a relatively stable construct, examining existential threat perceptions, which are more susceptible to change, as a process variable may open the possibility of fostering political change through changes in threat perception. To validate the notion of existential threat as a process variable we compare this model to an alternative model in which threat is the predictor.

### Method

#### Participants

A community convenience sample of 170 Israeli participants, 76 men and 89 women (five participants did not report their gender) ranging in age from 17 to 62 (*M* = 29.5, *SD* = 9.9) were recruited on commuter trains running between Tel-Aviv and Haifa.

#### Materials and Procedure

Research assistants handed out questionnaire packets to commuters who agreed to participate (58% response rate). The questionnaire packet included the 20-item MET scale [α = 0.86 for SA, α = 0.82 for PA, α = 0.86 for PV, and α = 0.86 for PD) and five items that measured out-group variability (based on Brauer and Er-rafiy, 2011: “Among Palestinians there are different kinds of people”; “Most Palestinians think alike” (R); “When I think about Palestinians I see them as different from each other”; “When I think about Palestinians I see all of them as bad (R)”; “all Arabs are the same” (R)]. These items were answered on a 7-point scale ranging from (1) “strongly disagree” to (7) “strongly agree” (α = 0.85). We also included five items that measured out-group communality (based on Saguy and Halperin, 2014: e.g., “Arabs and Jews have much in common”), and out-group trust [based on Nadler and Liviatan, 2006: e.g., “you can trust Arabs,” “you have to be careful and not rely on Arabs” (R)]. These items were answered on a 7-point scale ranging from (1) “strongly disagree” to (7) “strongly agree” and were combined into one score because of the acceptable reliability between items (α = 0.74).

The packet also included a 12-item scale measuring participants’ support for compromise and a political solution to the conflict with the Palestinians [e.g., “it is pertinent to resolve the conflict with the Palestinians”; “We must give the territories back to the Palestinians to resolve the conflict”; “United Jerusalem must remain the capital of Israel” (R)]. These items were scored on a 7-point scale ranging from (1) “strongly disagree” to (7) “strongly agree” (α = 0.87).

Then, participants completed a demographic questionnaire, and a single-item in-group identification question: “to what extent do you identify with other Israeli Jews?” answered on a 7-point scale ranging from (1) “very little” to (7) “very much.”

### Results and Discussion

To examine the pattern of associations between the main study measures – personal death (PD); past victimization (PV); collective physical annihilation (PA); collective symbolic annihilation (SA), in-group identification, and attitudes toward the Israeli–Palestinian conflict – we conducted a series of Pearson correlations. Correlations coefficients are presented in **Table 4**.

**Table 4:** Correlation Coefficients between Study 3’s Main Measures.

		1	2	3	4	5	6	7	8
1	Personal death	–							
2	Past victimization	0.34ˆ***	–						
3	Physical collective annihilation	0.73ˆ***	0.23ˆ**	–					
4	Symbolic collective annihilation	0.21ˆ**	-0.12	0.43ˆ***	–				
5	Ingroup identification	-0.01	0.23ˆ***	-0.03	-0.18ˆ*	–			
6	Political violence	-0.01	-0.12	0.03	0.15ˆ*	0.01	–		
7	Territorial compromise	-0.30ˆ***	-0.39ˆ***	-0.16ˆ*	0.35ˆ***	-0.24ˆ***	0.09	–	
8	Communality and trust of Palestinians	-0.25ˆ**	-0.19ˆ*	-0.10	0.37ˆ***	-0.53ˆ***	0.09	0.64ˆ***	–
9	Palestinians as a homogeneous group	0.18ˆ*	0.27ˆ**	0.15ˆ*	-0.11	0.43ˆ***	0.06	-0.45ˆ***	-0.59ˆ***

*^∗^*p* < 0.05, ^∗∗^*p* < 0.01, ^∗∗∗^*p* < 0.001.*

The analyses indicated that higher scores on PD, PV, and PA relate to lower support of territorial compromise, and greater perceptions of the Palestinians as homogenous. PD and PV were also related to less feelings of trust toward the Palestinians. In contrast, higher scores on SA were linked to greater support of territorial compromise and greater trust of the Palestinians. Consistent with this pattern, SA was also associated with lower in-group identification, whereas PV with greater in-group identification. These associations corroborate the findings of Study 2.

**FIGURE 1:**
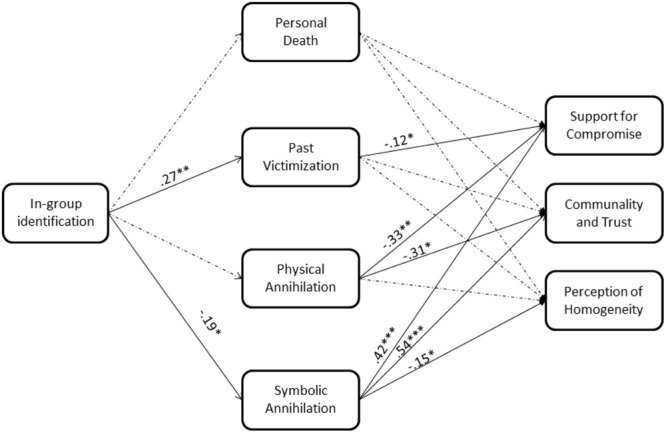
**Multidimensional existential threat (MET) factors – personal death, past victimization, physical collective annihilation, and symbolic collective annihilation – mediate the link between Israelis’ in-group identification, exposure to political violence, and their political attitudes on the Israeli–Palestinian conflict (Study 3)**.

Next, we examined whether the four facets of existential threat – PD, PV, PA, and SA – mediate the link between participants’ in-group identification and their attitudes toward the Israeli–Palestinian conflict. The specific attitudes measured in this study were: (a) support of territorial compromise with the Palestinians; (b) Feelings of communality and trust; and (c) perceiving the Palestinians to be a homogeneous group. To this end, we conducted a multiple mediation analyses using MPlus 6.1 (Muthén and Muthén, 1998-2010) SEM software, in which the measure of in-group identification served as the predictor, the MET factors served as the mediators (thereby assessing the unique contribution of each MET factor while controlling for the other three), and attitudes toward the Palestinians served as the outcome measures. Significance of the mediation paths were estimated using bias-corrected bootstrap analysis with 5,000 resampling. **Figure 1** presents these findings. The model had excellent fit to the observed data, χ^2^_(1)_ = 0.40, *p* = 0.53, *CFI* = 1.00, *TLI* = 1.00, *RMSEA* = 0.00.

The analyses indicated that Israeli in-group identification was associated with lower support of compromise [*b* = -0.37, *SE* = 0.08, *t* = 4.49, *p* < 0.001, *95% CI for b* (-0.53, -0.21)], greater perceptions of the Palestinians as homogenous [marginally significant; *b* = 0.15, *SE* = 0.08, *t* = 1.83, *p* = 0.069, *95% CI for b* (-0.04, 0.27)], and less trust of the Palestinians [*b* = -0.20, *SE* = 0.10, *t* = -2.02, *p* = 0.043, *95% CI for b* (-0.40, -0.01)].

These effects were significantly mediated by SA and PV. Specifically, in-group identification was associated with greater concerns over PV [*b* = 0.35, *SE* = 0.10, *t* = 3.53, *p* < 0.001, *95% CI for b* (0.15, 0.54)] and lower concerns over SA [*b* = -0.23, *SE* = 0.09, *t* = -2.61, *p* = 0.009, *95% CI for b* (-0.40, -0.06)]. Greater PV [*b* = -0.13, *SE* = 0.06, *t* = -2.12, *p* = 0.05, *95% CI for b* (-0.26, -0.02)] and lower SA [*b* = 0.40, *SE* = .09, *t* = 4.24, *p* < 0.001, *95% CI for b* (0.23, 0.58)] were linked with lower support for territorial compromise. Greater SA was also linked with greater trust of the Palestinians [*b* = 0.53, *SE* = 0.11, *t* = 4.92, *p* < 0.001, *95% CI for b* (0.34, 0.76)]. PA did not mediate the link between in-group identification and political attitudes, but was associated with lower support for compromise [*b* = -0.30, *SE* = 0.11, *t* = -2.71, *p* = 0.007, *95% CI for b* (-0.49, -0.06)], and lower trust of the Palestinians [*b* = -0.27, *SE* = 0.12, *t* = -2.28, *p* = 0.022, *95% CI for b* (-0.50, -0.04)]. Using SEM, we also conducted an alternative model in which in-group identification served as a moderator and not as a predictor. This model had poor fit to the observed data, χ^2^_(20)_ = 2233.98, *p* < 0.0001, *CFI* = 0.00, *TLI* = -13.15, *RMSEA* = 0.83. Overall, our model explained 32.5% of the variance in support for territorial compromise with the Palestinians, 24.7% of the variance in feelings of communality and trust, and 9.9% of the variance in perceiving the Palestinians to be a homogeneous group.

These results partially support our predictions, and are in keeping with the associations found in Study 2. Specifically, results suggest that among Israelis, different existential threats are associated with different political attitudes, such that symbolic threats relate to greater openness to the out-group and a peaceful political orientation, whereas physical threats relate to rejection of the out-group and greater belligerence. SA significantly mediated the relationship between in-group identification and political attitudes, but in line with Study 2, PA did not. Rather, PV which constitutes the memory of the threat of annihilation the group faced in the past mediated this relationship, indicating, perhaps, that the shadow of history looms large on this population. It is notable that PD was significantly associated with belligerent political attitudes as TMT would predict (Hirschberger and Pyszczynski, 2010), but after controlling for the other three existential threats, the association between PD, in-group identification and political attitudes was non-significant. In sum, the results of this initial study contribute to our understanding of why Israeli in-group identity is associated with belligerent and non-peaceful attitudes, and also suggest that a focus on Israel’s future identity, and not its traumatic past, is associated with more pragmatic and peaceful attitudes.

## Study 4

Study 3 constitutes a first attempt to examine whether qualitatively different existential threats are associated with different political outcomes. This study confirms the distinction between physical and symbolic threats, found in Studies 1 and 2, and also highlights the role of the temporal dimension (i.e., PV) in explaining Israeli political attitudes such that a forward looking perspective is associated with a motivation for reconciliation, whereas a past-focused orientation is associated with greater hostility toward the Palestinians. Study 4, conducted immediately following the 2014 Israel–Gaza War, had the following goals: (a) Examine whether existential threats mediate the relationship between a chronic ideological variable (i.e., political orientation) and political attitudes relevant to a specific context demonstrating the stability of the effects across time and across different intergroup contexts (peaceful vs. war time); (b) Measure a different set of outcome variables that focus directly on the 2014 war between Israel and Hamas in Gaza. Specifically, we were interested in tapping attitudes toward the conflict in Gaza along with general attitudes toward peace. In the current study, we also examined reactions toward in-group criticism of the war to see whether existential threats may underlie intolerance within the group; and (c) Examine whether political orientation, which is highly associated with in-group identification (e.g., Pedahzur and Canetti-Nisim, 2004) could be used as a predictor of existential threats and specific political attitudes in this study.

### Method

#### Participants

A near-representative sample of 512 Jewish–Israeli participants, 270 men (53.1%) and 242 women (46.9%) ranging in age from 18 to 79 (*M* = 41.83, *SD* = 15.55) were recruited through the Midgam Project^3^ – an online panel consisting of over 110,000 participants representing every geographic and demographic sector of Israel within the 1967 borders. Nine participants did not complete the questionnaire and were not included in analyses, leaving us with a sample of 503 participants. Participants who entered the study on the Midgam website were redirected to a Qualtrics platform using Interdisciplinary Center (IDC) Herzliya’s research management system.

#### Materials and Procedure

Following a consent form, and a brief introduction to the study that was presented as a survey on social and political issues, participants first completed the 20-item MET scale (α = 0.85 for SA, α = 0.83 for PA, α = 0.83 for PV, and α = 0.77 for PD). Then, they completed a five-item scale that tapped support for the War on Gaza (“It was wrong to end the operation prematurely”; “ending the operation without a clear victory is a sign of weakness”; “we should have conquered the entire Gaza Strip, even at a high cost”; “there was no point in continuing the operation in Gaza once the terror tunnels were destroyed”(R); “I am frustrated that the operation ended without defeating Hamas”). These items were answered on a 7-point scale ranging from (1) “strongly disagree” to (7) “strongly agree” (α = 0.82).

Next, participants completed an eight-item scale tapping support for diplomatic solutions to the War on Gaza (“It is an illusion to believe that Abbas can be our partner against Hamas”(R); “It is possible to create a new reality in Gaza with the help of Israel’s allies”; “Rehabilitating the Gaza Strip in exchange for its’ demilitarization is a good idea”; “The problem with Gaza can be solved only with military might and not with diplomacy”(R); “If Abbas takes control of the situation in Gaza it will improve the present reality in Israel”; “The future will be better due to diplomatic efforts to change the current situation”; “The belief that Abbas and Egypt are our partners in changing the situation in Gaza is a dangerous delusion”(R); “The siege on Gaza should be lifted to give the Palestinians some breathing space”). These items were answered on a 7-point scale ranging from (1) “strongly disagree” to (7) “strongly agree” (α = 0.80).

Following the diplomacy scale, participants answered a 9-item scale tapping support for a comprehensive peace agreement between Israel and the Palestinians (e.g., “The operation in Gaza shows that the conflict with the Palestinians can only be resolved by negotiations”; “The land-for-peace framework is the right way toward a better future”; “Real peace between Israel and the Palestinians is possible”; “The Palestinians will never stop fighting us, even if we agree to all of their demands”(R). These items were answered on a 7-point scale ranging from (1) “strongly disagree” to (7) “strongly agree” (α = 0.90).

The final four-item scale tapped attitudes toward criticism of the war expressed by in-group members (“Criticism of the war by the Israeli press hurt Israel”; “At such hard times we need to stay united and not express criticism”; “Israelis who criticize the Israel Defense Forces are not loyal citizens”; “critical discourse during the war is essential and may save leaders from making costly mistakes”(R). These items were answered on a 7-point scale ranging from (1) “strongly disagree” to (7) “strongly agree” (α = 0.80). After completing these questionnaires, participants entered their demographic information, including a question on their political orientation ranging from (1) “extreme left” to (7) “extreme right,” were electronically debriefed and thanked.

### Results and Discussion

To examine the pattern of associations between the main study measures – personal death (PD); past victimization (PV); collective physical annihilation (PA); collective symbolic annihilation (SA), political orientations, and attitudes on the 2014 War on Gaza – we conducted a series of Pearson correlations. Correlations coefficients are presented in **Table 5**. The analysis indicated that PD, PV, and PA were significantly associated with support for the war on Gaza. SA was negatively associated with support for the war, and was positively associated with peace and diplomacy as well as with tolerant attitudes toward in-group criticism. PV and PA were negatively associated with support for peace and diplomacy and associated with negative attitudes toward in-group criticism.

**Table 5:** Correlation coefficients between Study 4’s main measures.

		1	2	3	4	5	6	7	8	
1	Personal death	–						
2	Past victimization	0.25ˆ***							
3	Physical collective annihilation	0.53ˆ***	0.18ˆ***						
4	Symbolic collective annihilation	0.15ˆ**	-0.26ˆ***	0.26ˆ***					
5	Political orientation	0.06	0.19ˆ***	0.09ˆ*	-0.36ˆ***				
6	Support for war on Gaza	0.18ˆ***	0.19ˆ***	0.26ˆ***	-0.21ˆ***	0.48ˆ***			
7	Support for diplomatic solutions	-0.04	-0.11ˆ**	-0.19ˆ***	0.21ˆ***	-0.52ˆ***	-0.48ˆ***		
8	Support for peace	-0.03	-0.19ˆ***	-0.11ˆ**	0.41ˆ***	-0.69ˆ***	-0.54ˆ***	0.71ˆ***	
9	Reactions to in-group criticism	0.08	0.23ˆ***	0.09ˆ*	-0.43ˆ***	0.46ˆ***	0.40ˆ***	-0.35ˆ***	-0.53ˆ***

*^∗^*p* < 0.05, ^∗∗^*p* < 0.01, ^∗∗∗^*p* < 0.001.*

**FIGURE 2:**
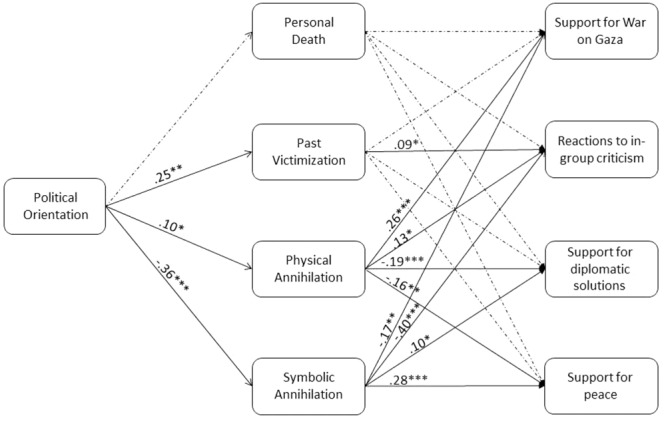
**Multidimensional existential threat factors – personal death, past victimization, physical collective annihilation, and symbolic collective annihilation – mediate the link between Israelis’ political orientation and their specific political attitudes on the Israeli–Palestinian conflict (Study 4)**.

Next, we examined whether the four facets of existential threat – PD, PV, PA, and SA – mediate the link between Israelis’ political views and their attitudes on solutions to the 2014 War on Gaza. The specific attitudes measured in this study were: (a) support for the war on Gaza; (b) support for diplomatic solutions; (c) support for peace between Israel and the Palestinians, and (d) reactions to in-group criticism. To this end, we conducted a multiple mediation analyses using SEM, in which the measure of political orientation served as the predictor, the MET factors served as the mediators, and attitudes toward the 2014 War on Gaza served as the outcome measures. Significance of the mediation paths were estimated using bias-corrected bootstrap analysis with 5,000 resampling. **Figure 2** presents these findings. The model had excellent fit to the observed data, χ^2^_(1)_ = 0.001, *p* = 0.97, *CFI* = 1.00, *TLI* = 1.00, *RMSEA* = 0.00.

The analyses indicated that right-wing views were associated with greater support for the war on Gaza [*b* = 0.64, *SE* = 0.05, *t* = 12.88, *p* < 0.0001, *95% CI for b* (0.55, 0.75)], lower support for diplomatic solutions [*b* = -0.54, *SE* = 0.04, *t* = -14.33, *p* < 0.0001, *95% CI for b* (-0.61, -0.46)], lower support for peace [*b* = -0.85, *SE* = 0.04, *t* = -22.41, *p* < 0.0001, *95% CI for b* (-0.93, -0.78)], and more negative reactions to in-group criticism [*b* = 0.64, *SE* = 0.05, *t* = 11.99, *p* < 0.0001, *95% CI for b* (0.53, 0.74)].

These effects were significantly mediated by SA, PA and PV. Specifically, right-wing views were associated with greater concerns over PA [*b* = 0.11, *SE* = 0.05, *t* = 2.18, *p* = 0.04, *95% CI for b* (0.007, 0.21)] and PV [*b* = 0.24, *SE* = 0.06, *t* = 4.45, *p* < 0.001, *95% CI for b* (0.14, 0.35)] and lower concerns over SA [*b* = -0.38, *SE* = 0.04, *t* = -9.20, *p* < 0.0001, *95% CI for b* (-0.46, -0.29)]. Greater PA [*b* = 0.13, *SE* = 0.05, *t* = 2.56, *p* = 0.010, *95% CI for b* (0.03, 0.23)] and/or PV [*b* = 0.09, *SE* = 0.04, *t* = 2.06, *p* = 0.045, *95% CI for b* (0.01, 0.18)] and lower SA [*b* = -0.40, *SE* = 0.06, *t* = -6.75, *p* < 0.0001, *95% CI for b* (-0.52, -0.29)] were linked with more negative reactions to in-group criticism. Greater PA and lower SA were linked with greater support for the war on Gaza [*b* = 0.24, *SE* = 0.06, *t* = 4.23, *p* < 0.0001, *95% CI for b* (0.13, 0.35) for PA and *b* = -0.14, *SE* = 0.06, *t* = -2.22, *p* = 0.026, *95% CI for b* (-0.25, -0.02) for SA] and peace [*b* = -0.14, *SE* = 0.05, *t* = -2.97, *p* = 0.003, *95% CI for b* (-0.23, -0.05) for PA and *b* = 0.26, *SE* = 0.05, *t* = 5.18, *p* < 0.0001, *95% CI for b* (0.16, 0.36) for SA]. Greater PA was also linked with less support for diplomatic solutions, *b* = -0.18, *SE* = 0.04, *t* = -4.06, *p* < 0.0001, *95% CI for b* (-0.26, -0.09). Using SEM, we also conducted an alternative model in which political orientation served as a moderator and not as a predictor. This model had excellent fit to the observed data, χ^2^_(1)_ = 0.05, *p* = 0.82, *CFI* = 1.00, *TLI* = 1.00, *RMSEA* = 0.00. None of the interactions were significant, however.

Overall, our model explained 31.4% of the variance in support for the war on Gaza, 32.0% of the variance in support for diplomatic solutions, 53.6% of the variance in support for peace between Israel and the Palestinians, and 33.7% of the variance in reactions to in-group criticism.

The results of Study 4 are in keeping with the pattern found in Study 3, using different predictors and outcome measures, and highlight the distinction between symbolic and physical existential threats found in the previous three studies. Specifically, Study 4 indicates that SA mediates the relationship between a left-wing political orientation and opposition to war, support for peace and diplomacy, and tolerance for in-group criticism. As in Study 3, PV mediated the association between a right-wing political orientation and political attitudes, but in Study 4 this mediating role was significant only for reactions to in-group criticism. In Study 4, unlike Study 3, PA played a significant role and mediated the relationship between political orientation and attitudes toward the war in the predicted direction, such that PA was associated with more support for the war, negative attitudes toward in-group criticism, and lower support for peace and diplomacy. Interestingly, in both studies 3 and 4, PD did not play a significant role after controlling for the other three existential concerns.

## General Discussion

The current research examined whether existential threat can be conceptualized as a multidimensional structure, and whether different existential threats are predictive of different political attitudes. The results of Studies 1 and 2 support our four-factor conceptualization of existential threats that are relevant to political conflict, and indicate that the four-factor solution fits the data better than other possible solutions. Study 2 established the convergent and discriminant validity of the MET, indicating that each MET factor is associated with different concerns and with different worldviews and ideologies. The results of Studies 3 and 4 provide further insight into how existential threats differ from one another in meaningful ways. Specifically, these two studies indicate that physical and symbolic existential threats carry a different meaning and are associated with different political outcomes. The correlational analyses revealed that perceptions of physical threat (personal, collective and past-oriented) are associated among Israeli Jews with less openness to the Palestinians (Study 3), less willingness to make compromises for peace (Studies 3 and 4), and at times of war (Study 4) with greater support for military action and rejection of diplomacy and political compromise. Perceptions of symbolic threat had a diametrically opposite effect and are associated with more openness and trust of the Palestinians (Study 3), greater support for peace and diplomacy (Studies 3 and 4), and less support for war. Across both studies, hawkish attitudes were associated primarily with past victimization (PV), but also with perceptions of collective physical annihilation (PA), and dovish attitudes were associated with perceptions of collective symbolic annihilation (SA).

### Existential Threat and Political Militancy

In the mediational analyses, however, the differences between the three physical existential threats (personal death, collective physical annihilation, and collective symbolic annihilation) emerged. In these analyses, we examined whether each existential threat significantly mediates the relationship between an ideological predictor (political orientation, in-group identity) and specific political attitudes, while controlling for the other three existential threats. This enabled us to assess the unique role of each threat that is untainted by its shared variance with the other threats. Results indicated that past victimization consistently predicted more belligerent and less peaceful attitudes toward the Palestinians in both studies. Perceptions of collective physical annihilation, on the other hand, had a significant mediating role only in Study 4 that was conducted immediately after a war with the Palestinians. When controlling for other existential threats, personal death did not play a significant role as a mediator.

The findings on past victimization are in keeping with previous research on exclusive framings of victimization (Vollhardt, 2012, 2015), and on the impact of the Holocaust on contemporary Jews, which shows that primes of the Holocaust increase hawkish political attitudes among Canadian Jews (Wohl and Branscombe, 2008), and that the Holocaust has become a central tenant of Israeli identity that influences the way Israelis perceive the conflict with the Palestinians (e.g., Klar et al., 2013; Canetti et al., in press; Hirschberger et al., in press).

The relationship that emerges from this research between a hawkish political orientation, perceptions of past victimization, and belligerent political attitudes is telling of the mindset of the Israeli right. Previous analyses of this mindset have described it as a siege mentality (Bar-Tal and Antebi, 1992), or an ethos of conflict (e.g., Bar-Tal, 1998), and the current research contributes to this literature by indicating that the temporal orientation of this mindset is almost exclusively backward focused. It is quite striking that the political attitudes of the Israeli right are predicated on a traumatic historical memory, and that this memory serves as the prism through which they understand Israel’s current problems and challenges. These results suggest that a collective post-traumatic reaction underlies this political ideology, and that to break the stalemate between Israel and the Palestinians it would be necessary to address this issue. In contrast, it appears that the ability of left-wing Israelis to compromise with the Palestinians involves the silencing of the ghosts of past traumas, such that the association of dovish political attitudes and support for peace is mediated by lower perceptions of past victimization. Hawkish Israelis did show some future-oriented concerns in Study 4 in the form of perceptions of collective physical annihilation. These perceptions, however, only further exacerbated the effects of past victimization and were associated with belligerent political attitudes.

One of the notable findings in this research is that after controlling for other existential threats, personal death was no longer significantly related to political outcomes. TMT has posited that the need to regulate personal death is a central human motivation, and terror management research has indeed shown that mortality salience influences a variety of social cognitions and behaviors (e.g., Greenberg and Kosloff, 2008). This research has also demonstrated that mortality salience typically leads to more belligerent political attitudes (e.g., Hirschberger and Pyszczynski, 2010). It is possible that in these studies, in which personal death was primed and participants were then exposed to questions and scenarios depicting intergroup conflict and collective level threats (e.g., Landau et al., 2004; Pyszczynski et al., 2006; Hirschberger et al., 2009), it was in fact collective existential threat, and not personal death, that was activated. Future research should attempt to tease apart the effects of personal and collective existential threat primes to determine which level of existential threat constitutes the active ingredient in the context of intergroup conflict.

### Existential Threat and Peaceful Attitudes

Symbolic existential threats consistently mediated the relationship between a liberal point of view and attitudes toward the Israeli–Palestinian conflict. At first glance, it may seem peculiar that those who are primarily concerned with the preservation of Jewish culture and identity are also more willing to make political compromises that would entail the surrender of territories that have historical meaning for Jews. This seeming inconsistency can be explained by the concern that Israel may lose its character as a Jewish state and a democracy if it does not cede control over the millions of Palestinians in the West Bank. The results of Study 2 support this explanation and show that the symbolic annihilation factor of the MET is significantly predicted by concerns over Israel’s future as a Jewish and democratic state, and higher estimates of risk to Israel’s identity as a democratic and Jewish country. Recently, a strategic assessment published by the Institute of National Security Studies (INSS: Michael, 2014) concluded that the changing demographics in Israel and the territories, and the lack of a political settlement in sight “may spell the end of the Zionist vision.” Thus, the perception that Jewish culture is in jeopardy is consistent with the desire to salvage the state from transforming into a different entity that might be less Jewish and/or less democratic.

It may also appear surprising that right-wing nationalistic Israelis show lower levels of concern over Israel’s symbolic existence. The right wing in Israel tends to deal with the Palestinian problem through minimization and denial. For instance, Naftali Bennett, A right-wing minister in the Netanyahu government has likened the Palestinian question to “shrapnel in Israel’s rear–end” (Verter, 2013), and the American–Israel Demographic Research Group, an influential right-wing organization^4^, rejects the official reports of Palestinian and Israeli demographics, and claims that Israel can maintain its Jewish and democratic character without any territorial compromises. Thus, hawkish political attitudes may be associated with lower concern over symbolic annihilation because right-wing Israelis feel secure about Israel’s identity, and deny the existence of any significant symbolic threat.

### Reexamining the Role of Threat in Political Attitudes

The results of the current research provide a more nuanced picture of the role of threat in political attitudes. Some have argued that threat invariably elicits more right-wing political views – a process termed conservative shift (Jost et al., 2003). Others have questioned whether threat always leads people to lean to the right or that perhaps in some cases threats may accentuate left-wing ideologies as well (Greenberg and Jonas, 2003; Castano et al., 2011). The results of our research offer a new way to address this question looking at threat as a multidimensional construct, and then the question is not whether threats induce conservative and liberal shift, but rather *which* threats are associated with shifts to the left or the right. Another way to look at this is that liberals and conservatives have different concerns and focus on different threats. For instance, the right-wing participants in the current research showed elevated concerns for physical collective threats, whereas left-wing participants placed the onus on symbolic collective threats. It seems, therefore, that the difference between left and right is predicated on the kind of threat and not the level of threat that is experienced.

Further, many models of threat and political militancy suggest that existential threat induces militant reactions via increased in-group identification (Canetti et al., in press). In the current study we show that existential threats can play a different role as a process variable that helps explain the link between in-group identity, political orientation and policy preferences. We show that this model has better fit with the data than an alternative model in which existential threats are the predictors. The multidimensional conceptualization of threat offered here can, therefore, not only provide more nuanced answers to existing questions, but can also help reconcile existing inconsistencies and debates in the literature.

### The Utility of a MET Model

The multidimensional model of existential threats presented here contributes to the understanding of the political attitudes of Israeli Jews, but can this model be extended to other cultures and other intergroup conflicts? The model clearly applies to the Israeli Jewish context wherein both physical and symbolic collective threats loom large, and there is also a long history of victimization that overshadows contemporary conflicts. We suggest, however, that our model may be relevant to understanding many other conflicts over existence and identity even when not all components of the model are necessarily present. The unique nature of each conflict will determine the impact of each specific existential threat. Thus, conflicts concerning other ‘small people’ (Abulof, 2015) – people that experience themselves as teetering on the edge of an abyss (e.g., Izidis, Kurds) – will likely involve significant concerns over collective physical annihilation, but in many other conflicts physical annihilation is not the major concern and the sense of threat centers on issues of symbolic identity. In some cases, existential threats may have different and even opposite effects from those found in the current research on Israeli samples. For example, whereas for Israelis the main collective symbolic concern is the transformation of Israel into a state with a Palestinian majority if a political solution to the conflict is not found, for Palestinians the main collective symbolic concern is the continuation of Israeli settlement activity in the West Bank that threatens to forestall their aspirations for statehood. Thus, we would expect symbolic collective concerns among Palestinians to be associated with greater belligerence (i.e., resistance to the occupation) and not with more peaceful attitudes due to the fundamental difference between their experience of symbolic existential threats, and the Israeli Jewish experience. These expected cultural and contextual differences notwithstanding, our model predicts that a multidimensional conceptualization of existential threats is relevant to all intergroup conflicts such that different threats will have different effects in all cultures and in all contexts.

Our conceptualization of existential threat may also help understand reactions to crises and conflicts far removed from the Palestinian–Israeli conflict such as individual and group differences in reactions to immigration in Europe. In this case, we would predict that physical concerns over increased terrorism would predict negative attitudes to immigration, but symbolic concerns over the transformation of Europe from an open pluralistic society to a more restricted society due to fear of terrorism would be associated with positive attitudes to immigration. The diametrically different responses of US President Barack Obama and then presidential candidate Donald Trump to the June 2016 Orlando attack signify these two concerns, with the former emphasizing the symbolic threat to American values, and the latter the physical threat of terrorism. European responses to immigration may also reflect a reaction to Europe’s dark history of racism and genocide. For some Europeans, the need to defend their group’s image may lead to negative attitudes to foreigners, but for others who wish to repent and change the ways of their group, perceptions of historical trauma may have the opposite effect and elicit altruism born of suffering (e.g., Staub and Vollhardt, 2008).

Conceptualizing existential threat as a multidimensional construct helps elucidate the meaning of different threats and their political implications, and also provides a framework that may help integrate the extant research on existential threats. The research presented here, however, is but an initial step toward understanding the domain of existential threats and their impact on political conflict. Although this research portrays a relatively consistent pattern of effects across four studies, it also has several shortcomings that should be noted. First, all studies employ a correlational design, and this limits our ability to draw causal inferences. Future research should attempt to replicate these effects using an experimental paradigm to determine the causal effect of specific existential threats on political outcomes. Second, this research was conducted on Israeli Jews for whom all of the existential threats in the MET model are relevant, and for whom existential threats have a specific meaning that may be different from the meaning it has for other groups. For instance, although we made clear *a priori* predictions about collective symbolic existential threats, we lacked a strong empirical basis on which to make these predictions. The results of the four studies presented here empirically substantiate these predictions on a specific population, but it remains unclear whether collective symbolic annihilation that is associated with peaceful attitudes in this population may have a different meaning, and therefore an association with different outcomes, in other cultures and in the context of other conflicts. It is likely that some cultures such as the Druze and Yazidis in the Middle East will show similar responses as Israeli Jews because they both experience historical victimization, and currently face the threat of physical (massive death due to war) and symbolic (forced conversions) collective annihilation due to the conflict in Iraq and Syria. In other cultures such as many European cultures, there may be less of a sense of historical victimization and more trepidation over the future identity of the group. Further, the current research, which is based only on existential threats that have already been identified in the literature, differentiates between different future threats (physical, symbolic), but collective victimization remains limited in scope and undifferentiated, as the focus is solely on historical trauma, and there is no distinction between concrete and symbolic historical traumas. Future research could examine whether ongoing collective victimization is captured by the symbolic and physical annihilation factors of the MET or stands as distinct. Research should also examine the difference between past threats to the existence of the group (i.e., genocide), and past events that may seem physical because of their violent nature, but never posed a threat to group existence, but to important group symbols of government, economic and military dominance (e.g., the 9/11 attacks). It is important to note that despite the distressing nature of this research, none of our participants expressed any distress during debriefing.

These limitations notwithstanding, our attempt to conceptualize and measure existential threats as a multidimensional construct has produced encouraging results indicating that existential threats play an important role in political cognition. Moreover, it appears that existential threat is not a unitary construct, and that to better understand the role of existential threat on political outcomes, it is prudent to consider the distinct implications of qualitatively different existential threats.

## Ethics Statement

This research was approved by the institutional review board (IRB) of the Interdisciplinary Center Herzliya. Participants signed an informed consent sheet prior to participation and were debriefed at the end of the procedure. No vulnerable populations were involved in this research.

## Author Contributions

GH developed the idea, and led the writing of the manuscript; TE-D conducted statistical analyses and helped edit the manuscript; BL and TS contributed to the research methodology and helped edit the manuscript.

## Conflict of Interest Statement

The authors declare that the research was conducted in the absence of any commercial or financial relationships that could be construed as a potential conflict of interest.

## Footnotes

1 http://www.winsteps.com

2 http://www.ipanel.com

3 http://www.midgam.com

4 http://www.israeldemographics.com
